# The emerging role of exosomal LncRNAs in chronic fatigue syndrome: from intercellular communication to disease biomarkers

**DOI:** 10.3389/fmolb.2025.1653627

**Published:** 2025-08-29

**Authors:** Lei Wang, Yujia Xu, Xiang Zhong, Guiping Wang, Zijun Shi, Can Mei, Linwanyue Chen, Jianbo Zhan, Jing Cheng

**Affiliations:** ^1^ School of Economics and Management, China University of Geosciences, Wuhan, China; ^2^ Institute of Health Inspection, Hubei Provincial Center for Disease Control and Prevention, Wuhan, Hubei, China; ^3^ School of Public Health, Wuhan University of Science and Technology, Wuhan, Hubei, China; ^4^ School of Medicine, Yangtze University, Jingzhou, Hubei, China

**Keywords:** exosomes, lncRNAs, chronic fatigue syndrome, association, biomarkers

## Abstract

Chronic fatigue syndrome (CFS) is a complex disease involving multiple systems throughout the body with unknown pathogenesis and is characterized by chronic fatigue. To date, no effective treatment for CFS has been found, as well as biomarkers for early identification of diagnosis. However, exosomes, a subpopulation of extracellular vesicles (EVs), are membranous vesicles secreted by cells into the surrounding environment, and long noncoding RNAs (LncRNAs) in EVs can mediate inter-organ and inter-cellular communication, which maybe associate with CFS. Therefore, this study aims to review the association between EV-LncRNAs and CFS, and to explore whether LncRNAs can be used as potential biomarkers for early identification and diagnosis of CFS, which put forward new ideas and a theoretical basis for the pathogenesis of CFS, as well as the identification of novel targeted therapies.

## 1 Introduction

CFS is a chronic, disabling condition characterized by persistent fatigue that is not easily relieved by rest (Luo et al., 2022).The symptoms of CFS tend to be widespread and overlap with many other diseases, including excessive fatigue, depression, muscle pain, sleep disorders, dysbiosis, cognitive disorders, neuroendocrine disorders, and immune dysfunction (Arron et al., 2024; Cvejic et al., 2016; Mensah et al., 2017; Rowe et al., 2017; Campen et al., 2021; Lakhan and Kirchgessner, 2010). Table 1 summarises the symptoms of CFS. The global prevalence of CFS ranges from 0.1% to 2.5% (Lim et al., 2020), and is more common in adults, with onset usually between 20 and 45 years old (Cortes Rivera et al., 2019). The prevalence of CFS in the United States ranges from 0.5% to 1.5% (Toogood et al., 2021), which is 1.5–2 times higher in women than in men (Lim et al., 2020; Jason et al., 1999) and in England from 1.47% to 2.99% among adolescents (Luo et al., 2022; Estévez-López et al., 2020). Lim et al. showed that the overall incidence of CFS was 0.77% in Korea and 0.76% in Japan (Lim and Son, 2021). The onset of CFS is associated with high stress day-to-day life conditions. With the rapid development of society and increasing daily pressures, the incidence of CFS is increasing, and an Australian study showed that the average annual cost of CFS was approximately $14.5 billion (Close et al., 2020). Mirin et al. showed that CFS was associated with a greater economic and disease burden than any other disease in the United States (Mirin et al., 2020). It not only brings heavy economic burden to patients, families and society, which but also causes huge mental burden to patients and caregivers. Thus, CFS has become a major public health problem that needs to be addressed urgently.

**TABLE 1:** Chronic fatigue syndrome (CFS) symptoms.

Symptom category	Specific symptoms	Key findings	Source
Core symptoms	Fatigue, cognitive dysfunction, post-exertional malaise (PEM)	Fatigue and PEM are core diagnostic criteria for CFS; cognitive dysfunction is associated with neuroinflammation	Holtzman et al. (2019); Fatt et al. (2020)
Neurocognitive symptoms	Decreased memory, inattention	Information processing speed slows down, possibly related to brain dysfunction	Fatt et al. (2020)
Immune and inflammatory symptoms	Increased levels of TNF-α, IL-6	Immune system abnormalities play a significant role in CFS symptoms	Cliff et al. (2019)
Sleep disorders	Insomnia, fragmented sleep, non-restorative sleep	Sleep quality is significantly correlated with the severity of fatigue	Castro-Marrero et al. (2018)
Pain symptoms	Muscle pain, ioint pain, headaches	Widespread pain is a common symptom of CFS, and pain management needs to be strengthened	Rowe et al. (2017)
Autonomic dysfunction	Orthostatic hypotension, abnormal heart rate variability	Autonomic dysfunction may be one of the important mechanisms of CFS symptoms	Ryabkova et al. (2024); Van Cauwenbergh et al., 2014
Mental health symptoms	Depression, anxiety	Mental health symptoms are associated with HPA axis dysfunction	Nater et al. (2008)
Gastrointestinal symptoms	Abdominal pain, bloating, irritable bowel syndrome (IBS)	Gut inflammation and microbiome abnormalities may be related to CFS symptoms	Lakhan and Kirchgessner (2010)
Post-exercise symptoms	Increased fatigue, pain, decreased cognitive function	Abnormal immune response after exercise may be one of the mechanisms of PEM.	Nijs et al. (2014)
Impact on quality of life	Decline in physical function, mental health, social function	CFS patients experience a significant decline in quality of life, requiring multidimensional intervention	Weigel et al. (2025)

The pathogenesis of CFS includes disturbances of the immune system, genetic and epigenetic alterations, dysregulation of the hypothalamic-pituitary-adrenal cortex(HPA) axis and hormones, and viral infections (Deumer et al., 2021). LncRNA is defined as RNA greater than 200 nucleotides in length that does not encode a protein, which is now thought to play important roles in a variety of cellular processes, including cell cycle (da Silveira et al., 2022), differentiation and proliferation (Lu et al., 2021), metabolism (biology) (Sirey et al., 2019), and diseases (Lu et al., 2023), such as autoimmune diseases (Bost et al., 2022; Lodde et al., 2020; Elhai et al., 2023) and cancer (Zhang, 2024; Xiang et al., 2024; Ma et al., 2022; Li et al., 2024). The regulation of LncRNA is multifaceted, and the up/downregulation of their expression has been implicated in multiple system abnormalities, including those affecting the immune and neuroendocrine systems. Moreover, there is also evidence that LncRNAs are associated with viral infections (Wang et al., 2020; Kesheh et al., 2022; Zhang et al., 2024a; Sarfaraz et al., 2023; Chen et al., 2025).

Given that the pathogenesis of CFS and the functions of EV-LncRNAs are not yet clear, we put forward a scientific hypothesis for the first time. That it is, LncRNAs not only altered in disease states (Zhang et al., 2019), but may also be involved in the occurrence and development of CFS. In addition, we suggest that EV-LncRNA should be included as a circulating biomarker for the early diagnosis of CFS. As demonstrated by the application of liquid biopsy in neuroblastoma (Jahangiri, 2024), if EV-LncRNA panels are realised for dynamic tracking of pathway activation, it will be of great significance for the early diagnosis, therapeutic selection, and recurrence of CFS, which is a chronic disease that involves multiple organs and systems. Therefore, this systematic review addresses the association of EV-LncRNAs with the pathogenesis of CFS. Firstly, we discussed the correlation between the two in terms of immune disorders, abnormal mitochondrial energy metabolism, neuroendocrine system dysregulation, and viral infections. Secondly, we elaborated on the changes of LncRNA profiles in CFS patients. Lastly, we summarized the potential prospect of the use of EV-LncRNAs as biomarkers for early diagnosis of CFS.

## 2 LncRNAs and EVs

### 2.1 The history of LncRNAs

**FIGURE 1:**
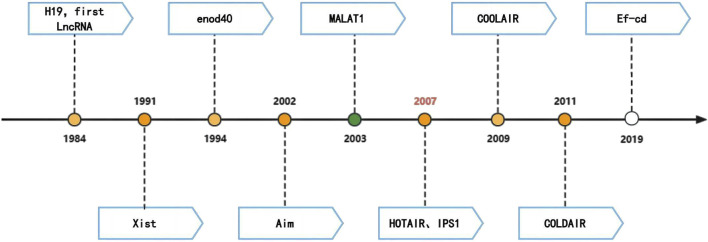
History of the LncRNAs.

The first discovery of LncRNA dates back to 1984, when LncRNA-H19 was identified in mammals by Pachnis et al. LncRNAs were initially considered as “noise” of genome transcription or by-products RNA of polymerase II transcription without a biological function. However, in 1991, Borsani et al. demonstrated the involvement of Xist in the regulation of X chromosome inactivation (Borsani et al., 1991). It was not until 1994, when the enod40 gene was found to likely play a role in plant development, acting as a “ribosome regulator” (Crespi et al., 1994). In 2003, Ji et al. predicted metastasis and survival in early non-small cell lung cancers using MALAT1 and thymic β4 (Ji et al., 2003). In 2007, Rinn et al. discovered the 2.2 kb-long HOTAIR in the human HOXC locus, which officially kicked off LncRNA research (Rinn et al., 2007) (Figure 1).

Over 100,000 human LncRNAs have been recorded to date (Uszczynska-Ratajczak et al., 2018), and LncRNA research has become an important area of research in biological sciences over the last decade. Namely, there have been over 50,000 publications with the keyword “Long noncoding RNA” and over 2,000 publications reporting validated LncRNA functions (Statello et al., 2021).

### 2.2 LncRNAs biogenesis, classification, and function

LncRNAs covered a large number of highly heteromerized transcripts that differed in biogenesis from their genomic origin (Chen and Kim, 2024). The main LncRNAs were transcribed by RNA polymerase II (Pol II), and could undergo splicing similar to that of mRNAs characterised by the addition of a cap [7- methylguanosine (m7G)] at the 5′ end and a polyadenylation at the 3′ end (polyA) (Statello et al., 2021). The vast majority of LncRNAs were derived from the nuclear genome (Statello et al., 2021). Based on the relative position of genomic LncRNA to neighbouring protein-coding genes, it could be divided into five categories: (1) Positive-sense LncRNA, overlapping with one or more exons of the encoding gene; (2) Antisense transcript product, partly or completely complementary to the transcript on the opposite strand; (3) Intron LncRNA, produced by introns of the gene; (4) The bidirectional transcription product, sharing the same promoters with protein-coding genes, but transcribed in the opposite direction; (5) Intergenic LncRNAs (LincRNA), transcribed independently by sequences located between protein-coding genes (Mercer et al., 2009; Rinn et al., 2012; Wang and Chang, 2011; Guttman and Rinn, 2012; Chodurska and Kunej, 2025).

Based on the location of LncRNAs and specific interactions with DNA, RNA, and protein, LncRNA could have the following functions: (1) It could regulate chromatin function, change the stability and translation of cytoplasmic mRNA, and interfered with signal transduction pathways (Statello et al., 2021); (2) It could act as a transcriptional regulator in the form of a cis or trans-acting element (trans), regulating gene expression (cis function) near its transcription site through various mechanisms, and targeting distant transcriptional activators or repressors or affecting gene transcription localization in cells (trans function) (Rinn et al., 2012; Ponting et al., 2009); (3) It could regulate organelles, with many LncRNA localized in specific organelles, such as exosomes and mitochondria (Ro et al., 2013; Kim et al., 2017). As exosomes were regularly released into the extracellular environment, exosome-localized LncRNAs could be secreted, and eventually entered recipient cells. In recipient cells, these LncRNAs could be involved in epigenetic inheritance, cell type reprogramming, and regulation of genome instability. Mitochondria-localized LncRNA could be encoded by nuclear and mitochondrial DNA, and was often associated with mitochondrial metabolism, apoptosis, mitochondria and the nucleus crosstalking (Statello et al., 2021; Zhang et al., 2024b).

### 2.3 The history of EVs

EVs are a variety of membranous structures secreted by cells that contain biologically active substances including proteins, lipids, and genetic substances (such as LncRNA) (Kim et al., 2017; Simeone et al., 2020). The study of EVs dates back to the mid-1940s, and although they were once considered the “trash bin” of our bodies, they are now regarded as the connecting bridge between cells (Chao et al., 2023). EVs travel through bodily fluids and transmit their molecular information in autocrine, paracrine, and endocrine manners (Krylova and Feng, 2023). EVs are also increasingly recognized as having a direct role in cancer and neuro-degenerative disease pathology. Therefore, the use of EVs as biomarkers of disease diagnosis and prognosis has gathered research interest (Kim et al., 2017; Urabe et al., 2020; Hill, 2019).

### 2.4 LncRNAs perform biological functions via EVs

**FIGURE 2:**
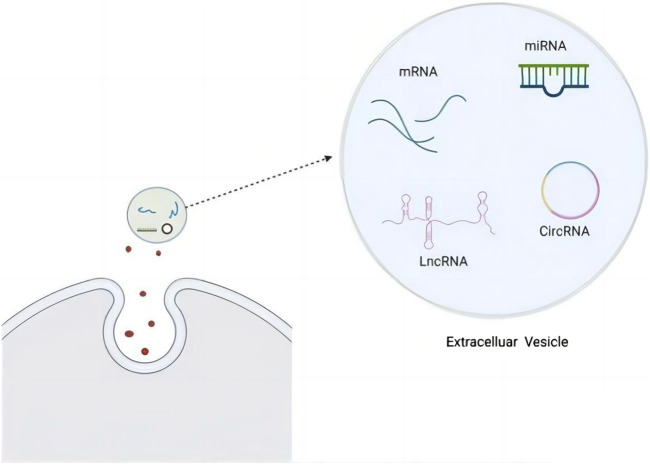
EV structure and content, including RNA (Kim et al., 2017).

In recent years, large amounts of LncRNAs have been found in peripheral blood, emulsions, urine, gastric fluid, and other bodily fluids (Yuan et al., 2020; Arantes et al., 2018). They have also been regarded as diagnostic cancer biomarkers (Zheng et al., 2021). These LncRNAs travel outside their cells of origin, and are selectively packaged into EVs. Subsequently, LncRNAs are transferred to proximal and distal recipient cells, inducing profound phenotypic changes (Li et al., 2020). EVs can be internalized by recipient cells via membrane fusion, receptor-dependent endocytosis, microcellular drinking, or phagocytosis (Hu et al., 2020).The structure and content of the EV is shown in Figure 2.These mechanisms determine the uptake of EVs with relative targeting and specificity. Cargo LncRNAs that are transported to recipient cells play their corresponding function, and participate in the occurrence and progression of disease, including the pathogenesis of CFS.

## 3 The association between EV-LncRNAs and CFS

**FIGURE 3:**
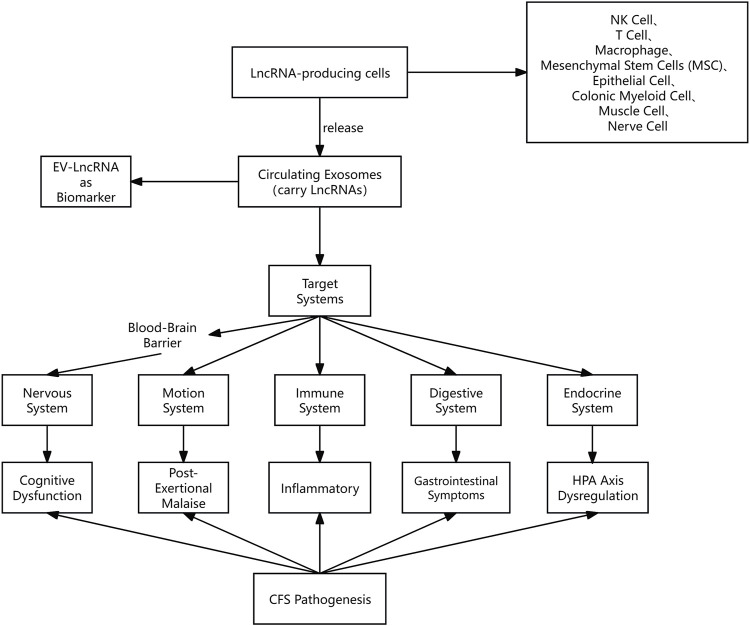
Proposed framework linking CFS pathogenic mechanisms to EV-LncRNA functions. This figure illustrates a paradigm in which exosomal LncRNAs act as intercellular messengers to coordinate the pathogenesis of CFS.Certain cells (e.g., T-cells, macrophages, neuronal cells, etc.) under pathological conditions of CFS may trigger the release of exosomal LncRNAs in response to tissue-specific stressors, thereby systematically propagating dysfunction across five target systems while generating detectable biomarker signatures in the circulation.

Given that the pathogenesis of CFS is multifaceted, involving immune dysregulation, epigenetic alterations, HPA axis dysfunction, and viral triggers, molecular regulators capable of integrating these pathways are of great interest. LncRNAs have emerged as key epigenetic regulators that orchestrate gene expression in a variety of cellular processes associated with CFS, including immune response, mitochondrial metabolism and neuroendocrine signalling. The integration framework is shown in Figure 3.

### 3.1 Mechanisms of EV-LncRNAs involved in cellular immune and neuroendocrine regulation

The immune system is composed of immune organs, immune cells, and immune active substances, which have several functions including immune surveillance, defence, and regulation. Attree et al. showed that although the pathogenesis of CFS is unclear, it is associated with abnormalities in the immune system (Arroll et al., 2014; Vu et al., 2024). Since 1987, there has been substantial evidence of impaired natural killer cell function (reduced cytotoxicity) in CFS patients, which is the most consistent immune finding in CFS (Caligiuri et al., 1987; Baraniuk et al., 2024a; Eaton-Fitch et al., 2019; Zhang et al., 2024c). When researchers measured Th1 and Th2 cell levels in CFS patients, they found that the Th1/Th2 cell ratio decreased, suggesting that a diminished Th1 cell-driven immune response may be an intrinsic immune abnormality associated with CFS (Capelli et al., 2010). In addition, plasma levels of TNF-α were higher in CFS patients compared with healthy controls (Kerr et al., 2001). Increased IL-10 levels were associated with somatic symptom severity (Bested and Marshall, 2015). It has also been reported that the levels of IL-8 and IL-13 are increased (Broderick et al., 2012). Other studies have shown that the HPA axis is decreased in patients with CFS (Rusin et al., 2022). Hormonal downregulation and hypometabolic states play an important role in fatigue, which may be related to cytokines (IL-1, IL-6). However, this mechanism is not clear and will not be reviewed here.

LncRNA Erythrocyte differentiation regulator 1 (Erdr1) has been shown to act as a key immune-modulator, playing an important role in various immune cells, including T cells (Kim et al., 2020), NK cells, and macrophages, etc. Erdr1 induces T-cell apoptosis, but enhances NK-cell toxicity (Shu et al., 2025). However, the specific mechanisms involved are still unclear and deserve further investigation. Linc-MAF-4 is a LncRNA with fine-specific expression in Th1 cells. Its main function is to inhibit the expression of MAF. Knockdown of linc-MAF-4 in human peripheral blood mononuclear cells (PBMCs) induced a shift in the differentiation of CD4^+^ T cells to Th2 cells. Over-expression of linc-MAF-4 on human naive CD4^+^ T cells promoted Th1 cell differentiation and inhibited Th2 cell differentiation. The above studies showed that linc-MAF-4 is an important molecule that promotes Th1 cell differentiation (Zhang et al., 2017). LncRNA GATA3-AS1 is an antisense LncRNA located on the antisense strand of the GATA 3 gene. Specifically expressed in Th2 cells, GATA 3A S1 can modulate the expression of GATA 3 and Th2-related effector cytokines IL-5 and IL-13 (Gibbons et al., 2018), thus affecting the differentiation of Th2 cells. In addition, in TOLL-like receptor (TLR)1/2-stimulated THP 1-derived human macrophages, LncRNA THRIL trans-regulates TNF-α expression by forming a complex with ribonucleoprotein (RNP) hnRNPL which acts on the TNF-α promoter (Li et al., 2014). NEAT 1 is another antisense LncRNA that stimulates IL-8 expression in Hela cells by binding to the splicing gene SFPQ, thereby inducing SFPQ translocation from the IL-8 promoter (Imamura et al., 2014).

#### 3.1.1 Summary of relevance

**FIGURE 4:**
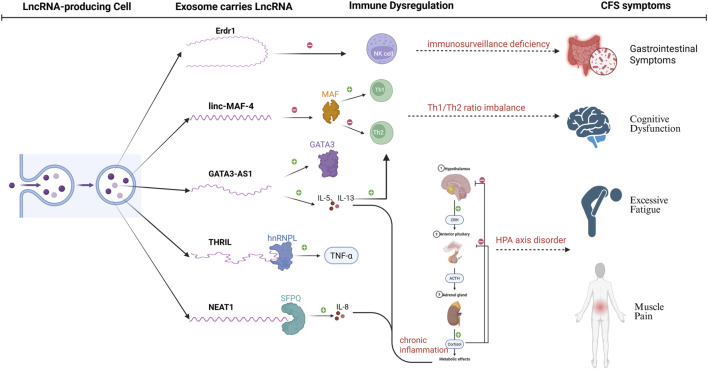
Regulatory roles of LncRNAs in immune dysregulation and pathogenesis of CFS. The figure illustrates the regulatory roles of LncRNAs (GATA3-AS1, linc-MAF-4, THRIL, NEAT1) in immune dysregulation and the pathogenesis of CFS. These LncRNAs modulate Th1/Th2 cell differentiation, cytokine production (e.g., TNF-α, IL-8), and NK cell function, leading to chronic inflammation and HPA axis suppression. These immune abnormalities contribute to core CFS symptoms, including cognitive disorders, excessive fatigue, and muscle pain.Solid lines: experimentally confirmed interactions; Dashed lines: hypothetical links to CFS pathology.

Based on previous studies, we have made several conjectures about the association of EV- LncRNA with CFS in the immune system (Figure 4).The following mechanistic hypotheses are extrapolated from LncRNA functions in general immunology studies. CFS-specific validation of exosomal LncRNA actions requires future experimental confirmation. (1) NK cell dysfunction and LncRNA modulation: Erdr1:it acts as an immunomodulator, enhancing NK cell cytotoxicity, while inducing T-cell apoptosis. Dysregulation of Erdr1 in CFS may impair NK cell function, reducing their ability to eliminate infected or abnormal cells and contributing to immune surveillance defects. (2) Th1/Th2 imbalance and LncRNA regulation:Linc-MAF-4:it promotes Th1 cell differentiation, while inhibiting Th2 cell differentiation by suppressing the transcription factor MAF. In CFS, reduced linc-MAF-4 expression may contribute to the observed Th1/Th2 imbalance, leading to diminished Th1-driven immune responses and a shift toward Th2-mediated humoral immunity. GATA3-AS1: As an antisense LncRNA, it enhances Th2 cell differentiation by upregulating GATA3 and Th2-related cytokines (IL-5, IL-13). Its overexpression in CFS could exacerbate Th2 polarization, further disrupting immune homeostasis. (3) Cytokine dysregulation and LncRNA involvement: THRIL: it forms a complex with hnRNPL to regulate TNF-α expression. Elevated THRIL levels in CFS may drive the increased TNF-α levels observed in patients, contributing to chronic inflammation and immune activation. NEAT1: By binding to the splicing factor SFPQ, NEAT1 promotes IL-8 expression. Its upregulation in CFS could enhance IL-8-mediated inflammatory responses, potentially exacerbating symptoms such as fatigue and pain. (4) Systemic immune dysregulation via EV-LncRNAs:it can influence immune cell differentiation and cytokine production by transferring regulatory signals between cells. In CFS, abnormal EV-LncRNA profiles may disrupt immune cell communication, leading to systemic immune dysregulation and chronic inflammation. (5) HPA axis suppression and LncRNA-Mediated inflammation:Chronic inflammation driven by LncRNA-mediated cytokine dysregulation (e.g., IL-1, IL-6) may suppress the HPA axis, leading to reduced cortisol levels. This hormonal downregulation could contribute to the hypo-metabolic state and fatigue characteristic of CFS. These mechanisms suggest that LncRNAs may serve as potential biomarkers or therapeutic targets for CFS, offering new insights into its pathogenesis and treatment. Further research is needed to elucidate the specific roles of LncRNAs and their potential for clinical applications.

### 3.2 Mechanisms of EV-LncRNAs involvement in mitochondrial dysfunction

CFS patients often present with an underlying energy deficit. Studies had shown that mitochondrial dysfunction might be an important cause of such deficit (Cortes Rivera et al., 2019; Syed et al., 2025). Some researchers had found that mitochondrial dysfunction (reduced ATP production) caused fatigue and post-exertional malaise. Moreover, it can cause overall metabolic abnormalities (Morris and Maes, 2014). Booth et al. studied the ATP profile of 138 CFS patients and 53 healthy controls, and showed that the majority of CFS patients had mitochondrial dysfunction, proposing that the ATP profile was associated with disease severity (Booth et al., 2012). The pathogenesis of CFS involves mitochondrial dysfunction, immune dysregulation, oxidative stress and abnormal energy metabolism (Wang et al., 2023; Bjørklund et al., 2020; Holden et al., 2020). In recent years, a large number of studies have demonstrated that LncRNAs play an important role in regulating mitochondrial function, which involves various aspects such as mitochondrial energy metabolism, oxidative stress, mitochondrial dynamics (fusion and fission), mitochondrial autophagy (mitophagy), and mitochondrial DNA (mtDNA) stability (Zhu et al., 2022; Chen et al., 2021; Niu et al., 2024). Therefore, LncRNAs may play an important role in the pathogenesis of CFS by regulating mitochondrial function.

HOXA11os is an LncRNA specifically expressed in the distal colon, and decreased levels of HOXA11os in colonic myeloid cells lead to complex I deficiency, oxidative phosphorylation dysfunction (OXPHOS), and the production of mitochondrial reactive oxygen species (mtROS) (Shmuel-Galia et al., 2023), which may contribute to intestinal dysfunction and inflammation in patients with CFS, if they have defective HOXA11os, and inflammation in the intestinal tract. Mitochondrial dysfunction leads to an increase in the production of ROS in the cell (LeFort et al., 2024), which induces oxidative stress and exacerbates mitochondrial damage (Veluthakal et al., 2024; Mo et al., 2024), whereas a study by Vicky et al. confirmed the role of the LncRNA ROSALIND as a novel ROS buffering system that protected mitochondrial translation from oxidative stress (Katopodi et al., 2025). Many studies have shown that oxidative stress is present in CFS patients (Bjørklund et al., 2020; Shankar et al., 2024; Skare et al., 2024; Sukocheva et al., 2022), so the dysregulation of ROSALIND without protection of dysfunctional mitochondria may be an important reason why fatigue is not alleviated in CFS patients. LncMtDloop, a LncRNA encoded within the D-loop region of the mitochondrial genome, plays a critical role in maintaining mitochondrial RNA levels and function. Although its precise mechanisms remain unclear, recent research has highlighted its potential involvement in neurodegenerative diseases. A study investigating the role of LncMtDloop in Alzheimer’s disease (AD) revealed that its expression levels are significantly reduced in AD patients. Experimental enhancement of LncMtDloop in AD model mice demonstrated a marked improvement in AD-like pathological and behavioral deficits (Xiong et al., 2024). Given that neurocognitive impairment is a shared characteristic between AD and CFS patients (Arron et al., 2024; Liu et al., 2024; Ferreira et al., 2024; Baraniuk et al., 2024b), we hypothesize that the downregulation of LncMtDloop in CFS patients may contribute to their cognitive deficits. However, this hypothesis requires further validation through extensive clinical studies to establish a definitive link between LncMtDloop dysregulation and cognitive impairment in CFS.

Glycolysis influences ATP production by providing pyruvate and NADH, supporting the mitochondrial TCA cycle and oxidative phosphorylation (Chandel, 2021). Glycolysis depends on mitochondrial pyruvate carboxylation and energy supply to maintain blood glucose levels (Hernández, 2021). The balance between glycolysis and gluconeogenesis is critical for maintaining mitochondrial function and cellular energy homeostasis, and its imbalance may lead to metabolic diseases (Guasch-Ferré et al., 2020; Dalga et al., 2023). Zhu et al. first proposed that a LncRNA termed glycoLINC could serve as a scaffold to assemble glycolytic enzymes into functional metabolons, enhancing glycolytic flux and ATP production to support cell survival under nutrient-deprived conditions (Zhu et al., 2022). In a separate study, it was demonstrated that LncRNA H19 modulates mitochondrial functions, including calcium homeostasis, ATP production, and ROS levels, as well as endoplasmic reticulum (ER)-mitochondrial coupling, by regulating the expression of the mitochondrial outer membrane protein VDAC1. Suppression of H19 was shown to enhance ER-mitochondrial coupling and significantly upregulate the expression of gluconeogenesis-related genes (Nandwani et al., 2024). Based on these findings, we hypothesize that alterations in the levels of glycoLINC and H19 in patients with CFS may disrupt the balance between glycolysis and gluconeogenesis. This imbalance could subsequently impair mitochondrial function, leading to metabolic dysregulation, which may underlie the metabolism-related symptoms observed in CFS patients.Table 2 summarises the association between CFS and LncRNAs in regulating mitochondrial functions.

**TABLE 2:** Association between CFS and LncRNAs: roles of LncRNAs in regulating mitochondrial function.

LncRNA	Function description	Regulation of mitochondrial processes	Potential association with CFS
glycoLINC	Serves as a scaffold for glycolytic enzymes, assembling functional metabolic complexes, enhancing glycolytic flux and ATP generation	Glycolysis, ATP generation	Glycolysis and ATP generation support cell survival under nutrient deprivation, potentially alleviating energy deficiency symptoms in CFS patients
H19	Regulates VDAC1 expression, affecting mitochondrial calcium homeostasis, ATP generation, and ER-mitochondrial coupling, upregulating genes related to gluconeogenesis	Mitochondrial calcium homeostasis, ER-mitochondrial coupling, gluconeogenesis	Regulation of gluconeogenesis and mitochondrial function may impact metabolic abnormalities and fatigue symptoms in CFS patients
HOXA11os	Specifically expressed in the distal colon, regulating complex I and mtROS generation	Oxidative phosphorylation, mtROS generation	Oxidative phosphorylation and mtROS generation may be involved in intestinal dysfunction and inflammation in CFS patients, exacerbating fatigue and metabolic disorders
ROSALIND	Serves as a ROS buffering system, protecting mitochondrial translation from oxidative stress damage	Oxidative stress, mitochondrial function protection	Disruption of ROSALIND may lead to mitochondrial dysfunction, exacerbating fatigue and oxidative stress symptoms in CFS patients
LncMtDloop	Maintains mitochondrial RNA levels and function, involved in mitochondrial gene expression regulation	Mitochondrial RNA stability, mitochondrial function	Downregulation in CFS patients may lead to mitochondrial dysfunction and neurocognitive impairment, related to cognitive symptoms of CFS

### 3.3 Mechanisms of EV-LncRNAs involvement in genetic susceptibility

Studies had suggested that CFS might be associated with genetic susceptibility. Van et al. compared dozens of adolescent CFS patients with healthy controls and their parents, and showed that children of women with CFS had similar mental illness conditions. The opposite was true for fathers (van de Putte et al., 2006). CFS is associated with several genetic pathways, including immune regulation and neurotransmission, inflammation and oxidative stress, and catecholamine pathways (Wang et al., 2017). These included changes in related genes such as TNF- α, IL-1, IL-4, IL-6, HLA, IFN-γ, and 5-HT (Wang et al., 2017). In addition, some CFS patients developed autoimmunity, which might be related to genetic backgrounds and disorders that promote B cell cloning, which in turn responded to self-antigens (Blomberg et al., 2018). Moreover, there were also methylation differences in the PRF 1 gene and at multiple CpG sites in T cells from CFS patients when compared with healthy controls (Herrera et al., 2018).

The effects of EV-LncRNAs on the immune system and the regulation of inflammatory factors had been described above. In addition to affecting immunological functions, EV-LncRNAs could also affect the genomic regulation of children from CFS patients. LncRNAs interacted with proteins and nucleic acids that regulated gene expression, facilitating solid, flexible and specific transcriptional and post-transcriptional control in the nucleus and cytoplasm (Herman et al., 2022). LncRNAs may have very important genetic and epigenic roles, indicating involvement in the regulation of altered genetic information in CFS patients. Nonetheless, there is a paucity of studies on the genetic aspects of LncRNA and CFS, and a large number of studies are needed to confirm the genetic association between them.

### 3.4 Mechanisms of EV-LncRNAs involved in viral infections

As early as 1985, Jones et al. reported for the first time the association between CFS (then called “chronic EBV infection syndrome”) and EBV infection. The study found that some CFS patients showed elevated EBV antibody titres, suggesting that EBV infection may be associated with the development of CFS (Winston et al., 1985). It has been demonstrated that human herpesvirus (HHV)-7, parvovirus B19, Borna disease virus (BDV), enterovirus, and coxsackie group B virus infections are risk factors for the development of CFS, with BDV being the most strongly associated (Hwang et al., 2023; Ariza, 2021). During the COVID-19 pandemic, many studies had shown that in the months following a SARS-CoV-2 infection, patients experienced immune, inflammatory, cardiovascular, intestinal, metabolic, and neurological changes which had some overlapping symptoms with CFS (Sukocheva et al., 2022; Vojdani et al., 2023), which suggested that there might be a potential correlation between the occurrence and progression of CFS and COVID-19.

LncRNAs have recently been described as key regulators of viral infections, with involvement in antiviral responses and virus-host interactions (Sarfaraz et al., 2023; Vierbuchen and Fitzgerald, 2021; Chen et al., 2022), some of which may be beneficial to the virus (Liu and Ding, 2017). This was confirmed by Cao et al. who found that LncRNA-BTX is upregulated by IRF3 -type I interferon-independent pathway after viral infection to help the virus evade clearance by the immune system, and also regulates the intracellular localisation of DHX9 and ILF3 by enhancing the interactions of RNA-binding proteins (RBPs) DHX9 and ILF3 with their partner proteins (JMJD6 and ILF2). Intracellular localisation of DHX9 and ILF3: facilitates viral replication by promoting the return of DHX9 to the cytoplasm while retaining ILF3 in the nucleus (Cao et al., 2023). IFN is a glycoprotein produced by viruses or other interferon inducers that has antiviral properties (Samuel, 2001), LncRNA-ISIR deficiency *in vivo* can lead to reduced IFN production, uncontrolled viral replication, and increased mortality (Xu et al., 2021). CHROMR, an LncRNA induced to be expressed in influenza A virus and SARS-CoV-2 infections, enhances the antiviral immune response by sequestering the interferon regulatory factor (IRF)-2-dependent transcriptional co-repressor, IRF2BP2, and promoting the expression of interferon stimulated genes (ISGs) (van Solingen et al., 2022). LncRNA-MALAT1 may contribute to antiviral responses through interactions with IRF 1, IRF 4, STAT 1, STAT 3, and STAT5A (Kesheh et al., 2022). Alternatively, Yang et al. showed that LncRNA modulates RIG -1 signalling, including the regulation of biological processes involved in COVID-19 and subsequent disease states (Yang et al., 2021). B cells infected by viruses can act on Th cells through the release of exosomes containing LncRNA. These can alter Th1 and Th2 cell differentiation, preventing cellular and humoral immunity from occurring normally. In addition, LncRNA may directly affect Th1 cell secretion of IFN-γ to activated other cells, such as epithelial cells, endothelial cells, fibroblastic, and glial cells. The latter express MHC-Ⅱ molecules and become non-professional antigen-presenting cells during EBV infection (Ruiz-Pablos et al., 2021) (Figure 6). Therefore, the regulatory role of LncRNAs in viral infections and their impact on immune responses, which suggests that LncRNAs may be involved in the pathogenesis of CFS, and that viral infections may participate in the onset and progression of CFS through LncRNA-mediated mechanisms.

## 4 EV-LncRNAs as potential biomarkers for the diagnosis of CFS

To date, diagnostic methods for CFS patients are scarce due to unknown pathogenesis and symptoms involving multi-system abnormalities. Therefore, an unbiased and specific biomarker is urgently needed to expedite the diagnosis and treatment of CFS patients. Activin B has been proposed as a CFS biomarker, but its use remains controversial (Gravelsina et al., 2021; Lidbury et al., 2017). A non-coding RNA (miRNA), has also been proposed as a biomarker to diagnose CFS. However, while changes in miRNA expression profiles are of great significance for the diagnosis of CFS, the lack of recruitment and the influence of age, gender, motor status, and other factors on the expression profile are limiting factors. As a result, the utility of miRNA is currently stagnant (Brenu et al., 2014; Cheema et al., 2020).

In recent years, more and more studies have shown that EV-LncRNA can be used as a biomarker for early diagnosis of diseases. By means of liquid biopsy, it has been found that the levels of LncRNAs change in infectious diseases such as sepsis (Li et al., 2022), tumours such as gastric cancer (Guo et al., 2023), Pancreatic cancer (He et al., 2024), breast cancer (Meng et al., 2024), gynecological diseases such as endometriosis (Shan et al., 2022), cardiovascular diseases such as atrial fibrillation (Kang et al., 2022), and neurodegenerative diseases such as Alzheimer’s disease (Canseco-Rodriguez et al., 2022) and autoimmune diseases such as rheumatoid arthritis (Wu et al., 2024), which is of great significance in early diagnosis of the diseases and long-term prognosis. In addition, Su et al. found that lncRNAs are stably present in exosomes of serum or urine. The mechanism may be that the membrane structure of exosomes can act as a protective membrane to protect these molecules from degradation (Su et al., 2021). Given the stability of lncRNAs in exosomes and the economy, simplicity, reproducibility, and noninvasiveness of the assay in serum and urine samples, we hypothesised that exosome-derived lncRNAs could also be used as clinical biomarkers for CFS. Yang et al. examined the expression characteristics of 10 LncRNAs in PBMCs of 44 CFS patients, and found that NTT, MIAT, and EMX2OS (three immune-related LncRNA) values were significantly increased. Moreover, NTT and EMX2OS expression levels were correlated with disease severity. Similarly, the downstream genes IFNGR1, NTT, and PBOV 1 were tested, and the NTT-IFNGR1 axis was found to play a role in CFS, although its mechanism was still unclear and further studies were needed (Yang et al., 2018).

## 5 Discussion

**FIGURE 5:**
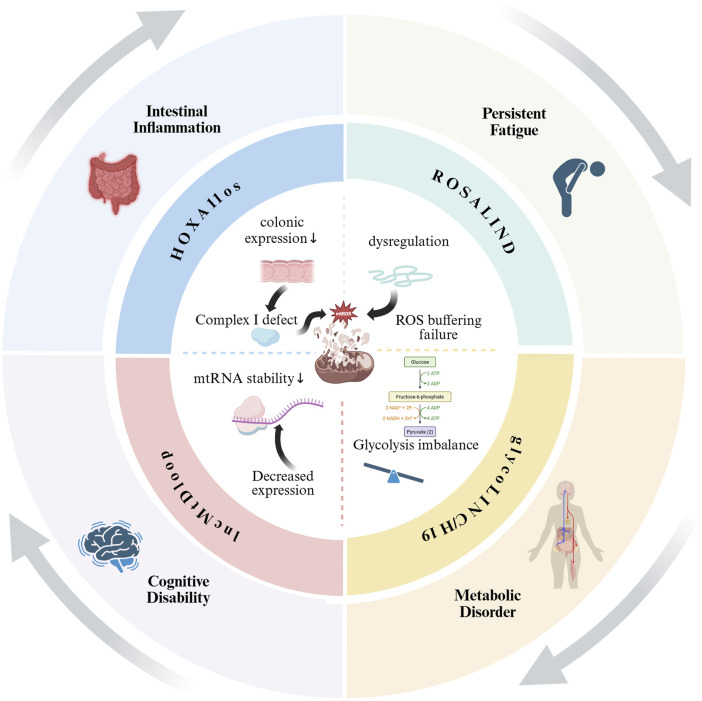
Circular diagram of LncRNAs-mediated mitochondrial dysfunction in CFS. Notes: This figure illustrates how dysregulated LncRNAs (HOXA11OS, ROSALIND, LncMtDloop, and glycoLINC/H19) contribute to mitochondrial dysfunction (center), driving key symptoms of CFS. HOXA11OS downregulation in the colon impairs Complex I, increasing ROS and intestinal inflammation. ROSALIND failure to buffer ROS worsens oxidative stress, while LncMtDloop reduction destabilizes mtRNA, linking to cognitive deficits. Altered glycoLINC/H19 disrupts glycolysis/gluconeogenesis, causing metabolic disorder. The circular layout emphasizes the vicious cycle between mitochondrial damage and clinical manifestations.

**FIGURE 6:**
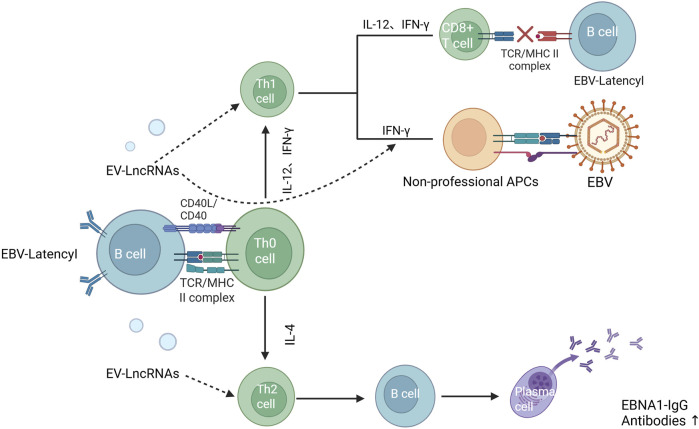
Effect of EV-LncRNAs on the antiviral immune response (Ruiz-Pablos et al., 2021). Notes: EBV-Latency1 B cells can affect the body’s cellular and humoral immune effects byreleasing exosomes that allow LncRNA to act on Th1 and Th2 cells, respectively. In addition, LncRNA can also directly affect the expression of IFN, which induces other cells, such as endothelial cells, epithelial cells, fibroblasts, and glial cells, to express MHC-II molecules and to become non-specialised antigen-presenting cells to be infected with EBV.

CFS is a complex chronic disease with an unknown mechanism, and involves multiple organs. In this study, we reviewed the possible pathogenesis of CFS, as well as the history of LncRNA discovery, functional classification, and roles in EVs. Next, we explored the association of EV- LncRNAs and CFS from an immunity, neuroendocrine, inflammation, mitochondrial dysfunction, and genetic susceptibility viewpoint, and proposed that LncRNA in PBMCs of CFS patients could be used as a potential diagnostic biomarker. However, the impact of patient heterogeneity required consideration. Our analyses suggest that EV-LncRNAs are molecular connectors linking the major etiological domains of CFS. As shown in Figures 4–6, specific LncRNAs (e.g., THRIL is associated with immune inflammation and glycoLINC with mitochondrial metabolism) functionally map to different pathological axes of CFS. This supports our initial hypothesis that LncRNAs provide an epigenetic layer that integrates the multifactorial origins of CFS and that exosome packaging enables the systemic propagation of dysregulation.

With regard to cellular immunity, we compared in detail the results of the present study with those of existing literature on the effects of EV-LncRNA on immune cell differentiation and function. For example, we found that the promotional effect of Linc-MAF-4 on Th1 cell differentiation observed was consistent with the results in the literature (Zhang et al., 2017), further confirming its importance in immunomodulation. However, for the role of certain LncRNAs (e.g., LncRNA-GATA 3-AS1) in immune regulation, the results of the present study differed from some of the literature. We delved into the possible reasons for these discrepancies, taking into account the heterogeneity of the participants, including factors such as age, gender, disease severity, and genetic background that might have an impact on LncRNA expression and function (Gibbons et al., 2018). While the Th1/Th2 ratio was decreased in CFS patients, a weakened Th1-driven immune response may be the underlying cause of their immune dysfunction. In addition, some cytokines, including TNF-α, IL-1, IL-6, IL-10, IL-8, and IL-13 had altered expression in CFS patients, and might impact CFS patients in three distinct ways. Firstly, they affected the differentiation and function of immune cells, playing an important role in the body’s immune response. Secondly, some of them could act on thermo-regulation centres, which explained why some CFS patients present with fever. Thirdly, they might degrade the HPA axis, abnormally secreting specific hormones (such as glucocorticoids, minerocorticosteroids, sex hormones), and affecting the normal functions and metabolism. While these cytokines could be regulated by LncRNAs, different LncRNA-regulated cytokines were distinct. This comparative analysis provided a more comprehensive understanding of the complex regulatory network of EV-LncRNAs in the immunopathogenesis of CFS.

With regards to mitochondrial function, ATP is an important substance in the human circulatory metabolism and plays a vital role in the energy supply of the body. Studies had shown that the ATP content in CFS patients was decreased, is correlated with disease severity, and could be used for the diagnosis of CFS (Booth et al., 2012). In terms of mitochondrial dysfunction, we carefully compared the expression changes and functions of LncRNAs (e.g., glycoLINC, H19, HOXA11os, ROSALIND) related to mitochondrial function identified with those found in the existing literature. It was showed that the roles of these LncRNAs in regulating mitochondrial function had certain similarities and correlations with findings in other disease models (Rackham et al., 2011). For example, the function of H19 in mitochondria was similar to that reported in other mitochondria-associated diseases, suggesting its general importance in maintaining normal mitochondrial function.Notably, the hypometabolic state of CFS patients is primarily characterised by impaired mitochondrial energy production, and enhanced gluconeogenesis may be a compensatory response of the organism to cope with the energy crisis.H19-mediated upregulation of gluconeogenic genes, while contributing to the maintenance of glycaemic homeostasis, may exacerbate the metabolic burden in the long term and further deteriorate mitochondrial function - in line with the vicious circle model we propose in Figure 5. Meanwhile, we also focused on certain unique findings in this study, such as the effect of specific LncRNAs on mitochondrial gene expression in CFS patients, which provided new clues for further investigation of the molecular mechanisms of mitochondrial dysfunction in CFS. These LncRNAs could have greater or lesser impact on the function of mitochondria, and affected the production of ATP *in vivo*, leading to some CFS patients presenting with symptoms of insufficient energy supply.

When considering genetic susceptibility, children of CFS patients also appeared to show similar symptoms. However, symptoms related to genetic inheritance remained to be verified. Based on the comparison with the results in the literature, we have proposed some new insights and research directions. For example, the association between viral infections and the pathogenesis of CFS, although it had been shown that a variety of viral infections were associated with CFS, the specific role of EV-LncRNAs remained to be explored. We speculated that certain EV-LncRNAs might be involved in the development of CFS by regulating the antiviral immune response of host cells, influencing viral clearance and persistent infection. In viral infections, such as EBV and Borna disease virus (BDV), viruses were risk factors for the occurrence and development of CFS (Hwang et al., 2023). In addition, many researchers had proposed that symptoms after COVID exposured partially overlap with CFS, and LncRNAs could affect antiviral effects via the regulation of IFN and the RIG-1 signalling pathway (Yang et al., 2021). It suggested that when the expression of some LncRNAs was abnormal, the immune response was affected and viral infection, which might cause CFS.

Finally, we summarized the experimental results of Yang et al. in the detection of LncRNAs in PBMCs of CFS patients and healthy controls, in order to propose EV-LncRNAs as a biological index of CFS diagnosis/prognosis (Yang et al., 2018). However, the use of this required extensive research to overcome current limitations EV-LncRNAs and patient heterogeneity. So, we suggested that future studies should carry out larger-scale, multicentre clinical studies to include more representative CFS patients and healthy controls, and at the same time record in detail the patients' clinical characteristics, disease severity, treatment history, and other information, in order to more accurately analyse the relationship between EV-LncRNA and CFS. We propose to address patient heterogeneity through subtype stratification, which can be broadly categorised as immune-dominant, metabolic-dominant, and neuroinflammatory, and to minimise pre-analytical variability through centralised biobanking (plasma processing within 2 h of blood draw). In addition, emerging technologies, such as single-cell RNA sequencing, can be combined to analyse EV-LncRNA expression in different cellular subpopulations, so as to gain a deeper understanding of its cell-specific role in the pathogenesis of CFS.

## 6 Conclusion

We first explored the correlation of EV-LncRNAs with CFS pathogenesis, and found LncRNAs potentially involved in CFS via immunological, neuroendocrine, mitochondrial, and viral factors. Changes in LncRNAs profiles may be crucial for CFS diagnosis and prognosis, which suggests that EV-LncRNAs maybe as a potential biomarker, and policymakers should support its development and integration into clinical practice. Early detection enables timely interventions like lifestyle changes, psychological support, and drug treatment, improving patient outcomes and reducing the social and economic burden of CFS.

## 7 Strengths and limitations

In our study, there are some highlights. Firstly, it is the first to systematically explore the potential role of LncRNAs in the pathogenesis of CFS, which reveals a previously unstudied molecular link between these two fields. Secondly, we comprehensively reviewed all the literature related to the association between LncRNA and CFS, including immune dysregulation, mitochondrial dysfunction, epigenetic regulation and inflammation control. We identified overlapping pathways that might connect them. Thirdly, by correlating scattered studies, we first put forward a scientific hypothesis that LncRNAs may affect CFS through mechanisms like neuroinflammation or metabolic stress, which provides a solid foundation for future experimental verification.

At same time, there are a few limitations in the study. First, although our study reviewed possible associations, no current studies have clearly proven a causality or strong correlation between specific LncRNAs and CFS. Second, Our bioinformatics inferences rely in part on public transcriptomic datasets, which have inherent biases: technical bias: batch effects of heterogeneous sequencing platforms may distort LncRNA quantification, and biological bias: underrepresentation of CFS subtypes in batch RNA sequencing masks patient-specific mechanisms (Liu et al., 2025). Effectively mitigating these biases is critical to advancing our diagnosis and treatment of CFS. Then, due to the scarcity of directly relevant literature, our analysis just relies on indirect evidence from related fields, such as LncRNAs in neuroimmunology or fatigue-associated diseases, which may cause selection bias or overlook relevant studies. Finally, although overlapping pathways, like NF-κB signaling and oxidative stress, support the interaction between LncRNAs and CFS, these connections remain hypothetical, which require functional validation *in vivo* or vitro research.
